# Intramedullary nailing versus proximal plating in the management of closed extra-articular proximal tibial fracture: a randomized controlled trial

**DOI:** 10.1007/s10195-014-0332-9

**Published:** 2015-01-15

**Authors:** Ramesh Chand Meena, Umesh Kumar Meena, Gopal Lal Gupta, Nitesh Gahlot, Sahil Gaba

**Affiliations:** 1 Department of Orthopaedics, SMS Medical College and Hospital, Jaipur, 302004 India; 2 Department of Orthopaedics, Postgraduate Institute of Medical Education and Research, Chandigarh, India

**Keywords:** Intramedullary nailing (IMN), Proximal tibial plate (PTP), Proximal tibial extra-articular fractures, Prospective trial

## Abstract

**Background:**

Extra-articular proximal tibial fractures account for 5–11 % of all tibial shaft fractures. In recent years, closed reduction and minimally invasive plating and multidirectional locked intramedullary nailing have both become widely used treatment modalities for proximal and distal tibial metaphyseal fractures. This study was performed to compare plating and nailing options in proximal tibia extra-articular fractures.

**Materials and methods:**

This randomized prospective clinical study was conducted on 58 skeletally mature patients with a closed extra-articular fracture of the proximal tibia treated with minimally invasive proximal tibial plating (PTP) or intramedullary nailing (IMN) by trained surgeons at a tertiary trauma center.

**Results:**

Postoperative hospital stay (*p* = 0.035), time to full weight-bearing, and union time (*p* = 0.004) were significantly less in the IMN group than in the PTP group, but there was no clear advantage of either technique in terms of operative time (*p* = 0.082), infection rate (*p* = 0.738), range of motion of the knee (*p* = 0.462), or degrees of malunion and nonunion.

**Conclusion:**

Both implants have shown promising results in extra-articular proximal tibial fractures, and provide rigid fixation that prevents secondary fracture collapse.

**Level of evidence:**

Level 2, randomized controlled trial.

## Introduction

Extra-articular proximal tibial fractures account for 5–11 % of all tibial shaft fractures [1, 9] and often result from high-velocity trauma. They lead to complex tissue injuries involving bone and surrounding soft tissues [1]. Conservative management of these fractures has often resulted in malunion, nonunion, rotational deformity, or stiffness of adjacent joints [2–4], so there has been a shift towards operative management of these fractures in recent times. However, the optimal method of surgically treating these fractures remains debatable. Options include intramedullary implant, half-pin external fixation, hybrid or thin-wire external fixation, plate fixation, or a combination of these techniques [5, 17]. In recent years, closed reduction with minimally invasive plating and locked intramedullary nailing have both become widely used treatment modalities for proximal and distal tibial metaphyseal fractures [6–8], despite the absence of any conclusive proof of the superiority of one modality over the other.

Due to the paucity of the relevant literature and the lack of conclusive evidence to guide the selection of treatment options in such cases, we designed this randomized controlled study (RCT) in order to compare the plating and nailing options in proximal tibia extra-articular fractures. We intended to compare these options in terms of operative time, duration of hospital stay, period of non-weight-bearing, degree of reduction, union rate, malunion rate, infection rate, and rates of other possible complications which could possibly affect decision-making in relation to such fracture patterns.

## Materials and methods

This randomized prospective clinical study was conducted on 58 patients with extra-articular fracture of the proximal tibia (OTA 41-A2/A3) treated with minimally invasive proximal tibial plating (PTP) or intramedullary nailing (IMN) by trained surgeons at a tertiary trauma care center in the Department of Orthopedics, SMS Medical College and Hospital, Jaipur, between January 2009 and December 2012. After excluding 14 patients who were lost to follow-up, a total of 44 patients were included in the final outcome analysis. Ethical committee approval was obtained, and patients were recruited once written informed consent had been provided.

Skeletally mature patients with closed proximal tibial metadiaphyseal fractures were included in this study. The proximal tibia was defined as the region extending from the articular surface up to 1.5 times the medial to lateral width of the articular surface [6]. Patients with metadiaphyseal tibial fractures with an intra-articular extension, tibial shaft fractures, open fractures, pathological fractures, and patients with multiple musculoskeletal injuries to the same or opposite lower limb were excluded from the study.

Patient allocation to groups was randomized by computer prospectively through the use of sequentially numbered opaque envelopes. Envelopes were opened inside the operating theater by a nurse who was blind to the allocation. Group A patients were treated with IMN and group B patients received PTP.

**Fig. 1:**
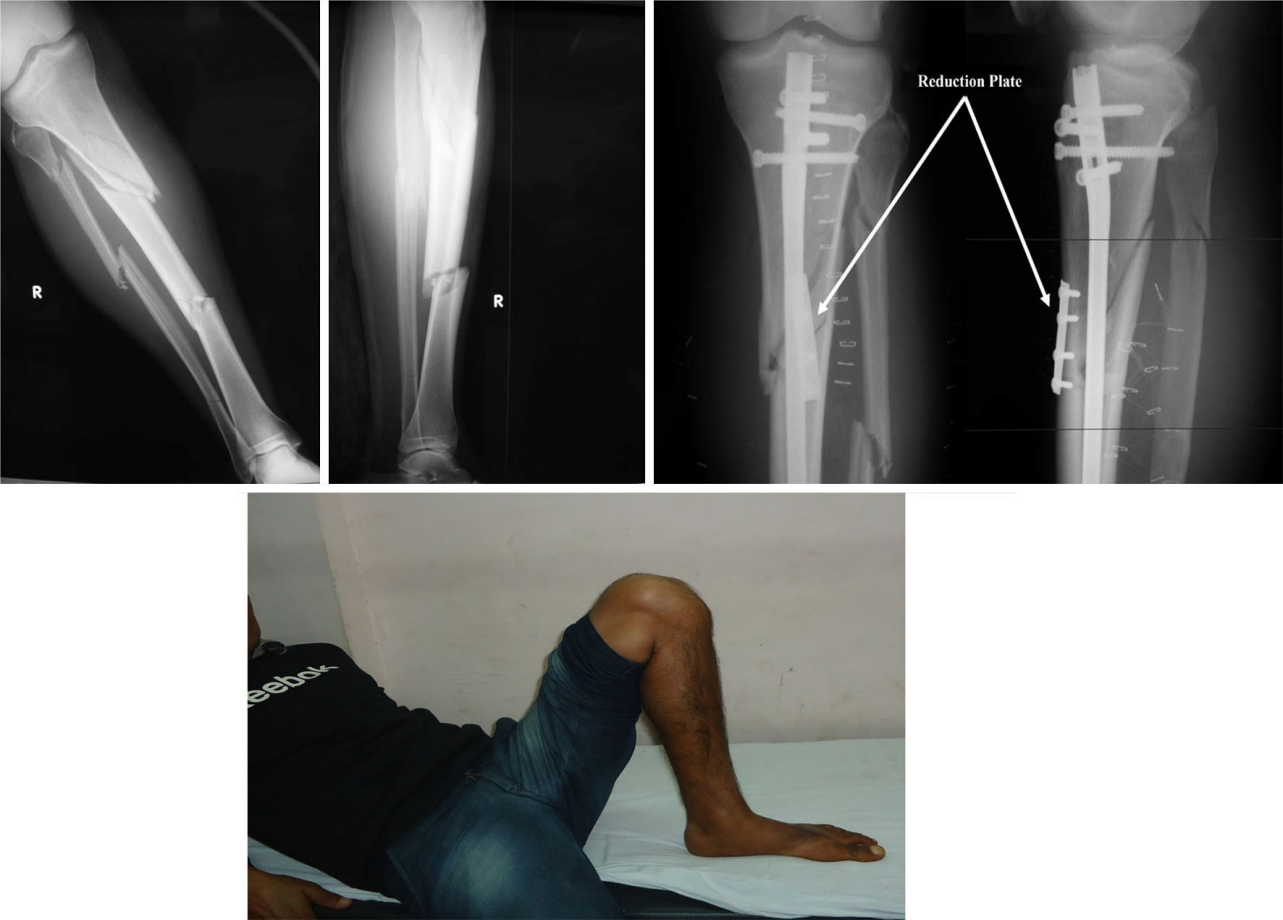
Patient with a segmental tibial fracture treated with expert tibial nail, showing a good range of motion of the knee postoperatively

The intramedullary nailing performed in group A was done by creating an entry point just medial to the lateral intercondylar eminence of the tibial plateau through a medial parapatellar approach. Temporary blocking screws, a reduction clamp, a reduction unicortical plate, or a universal fixator was used to achieve reduction and removed after fracture fixation, except for the reduction unicortical plate when used with a reamed intramedullary tibial nail. The intramedullary nail used had a proximal Herzog band and four multilevel, multiplanar, and multidirectional screws (Expert Tibial Nail, Synthes, Zuchwil, Switzerland) (Fig. 1).

**Fig. 2:**
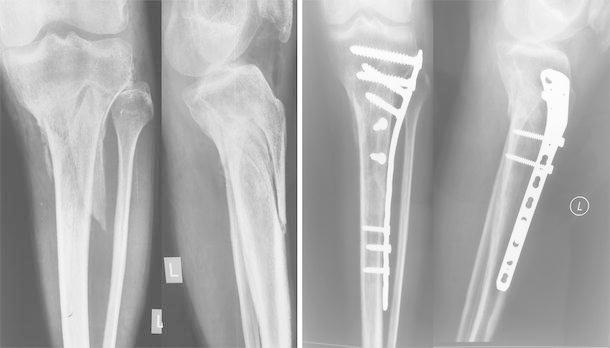
Preoperative and postoperative radiographs of a patient treated with plating

Patients in group B were treated by minimally invasive PTP using curvilinear incision over the lateral aspect of the proximal tibia. Indirect reduction was achieved using axial traction and/or the application of a reduction clamp or distractor. Internal fixation was then achieved with a proximal tibial lateral locking compression plate (LCP). A minimum of three screws were used on both sides of the fracture, and plating was done using a minimally invasive technique (Fig. 2).

Postoperatively, patients in both groups were given intravenous third-generation cephalosporin antibiotics for 3 days. Ankle pumps and isometric quadriceps strengthening exercises were started on the first postoperative day, followed by active and assisted knee bending on the second postoperative day. Partial weight-bearing was allowed from the second postoperative day, depending upon the stability of the construct, whereas full weight-bearing was allowed only after complete clinical and radiological union.

All patients were followed up at 2 and 6 weeks, 3 and 6 months, and 1 year postoperatively. Both the immediate postoperative and the final follow-up radiographs were compared to assess the accuracy of reduction and final alignment. Measurements were performed for coronal (varus and valgus) and sagittal (procurvatum and recurvatum) plane deformities using the measuring technique described by Freedman and Johnson [9]. In AP view, varus/valgus deformity was evaluated by measuring the angle between the lines drawn perpendicular to the proximal and distal tibial articular surfaces. In lateral view, the procurvatum/recurvatum was measured similarly and 8° of posterior slope was subtracted. Malreduction was defined as a deformity of >5° in any plane. Rotational alignment, shortening, and knee ROM were assessed clinically [9, 10]. The fracture was considered united if three or more cortices were continuous on two radiographic views. Nonunion was defined as three consecutive months of X-rays that did not show progressive healing.

All data were entered into a pro forma. The statistical analysis was performed by an independent statistician using the Statistical Package for the Social Sciences (SPSS version 22.0; SPSS, Chicago, IL, USA). The chosen level of significance was *p* < 0.05. The two groups were compared with respect to age, sex, operating time, hospital stay, infection rate, fracture union time, angulation of the fracture, and the knee range of motion. The parameters were compared between the groups. A paired-sample *t* test was used for the interval data (age, operating time, length of hospital stay, fracture union time, postoperative angulation, and range of motion of the knee).

## Results

Out of a total of 58 patients, 14 (6 in the IMN group, 8 in the plating group) were excluded from the study as they were absent for follow-up, meaning that 44 patients were included in the final outcome analysis. Preoperative characteristics including age, sex, classification, mode of injury, and time period from injury to operation were comparable in both groups (Table 1).

**Table 1:** Comparison of the demographic and postoperative data for both groups

	IMN group (group A)	PTP group (group B)	*p* value
Sex			
Male	14	18	0.961
Female	5	7	
Age	39 (18–65)	36 (19–62)	0.525
AO/OTA classification (OTA 41-A2/A3)	10/9	10/15	0.405
Operative time (h)	81.57 (60–110)	87.91 (60–120)	0.082
Hospital stay (days)	4.1 (2–8)	5.3 (3–10)	0.035
Union time (weeks) or time required before full weight-bearing (weeks)	18.26 (10–30)	22.84 (16–34)	0.004
Infection	0	2	0.738
Malalignment			
Coronal plane	2.77 (0–7)	2.08 (0–8)	0.296
Sagittal plane	2.57 (0–8)	2.19 (0–9)	0.415
Range of motion of knee	119.7 (90–150)	115.2 (80–150)	0.462
Delayed union/nonunion	2/1	0/1	0.849

Postoperative hospital stay, time period to full weight-bearing, and union time were significantly less in the IMN group as compared to the PTP group (Table 1). Surgical site infections (SSIs) were seen in two patients in the PTP group, one of which was resolved with debridement while the other necessitated implant removal due to infection.

Delayed union occurred in two patients in the IMN group, for which dynamization was performed by removing the distal screw. One case in the nailing group presented nonunion, which ultimately required exchange nailing with bone grafting and fibular osteotomy. There was nonunion in one patient in the PTP group; bone grafting was done in that case, which eventually led to fracture healing.

The alignment of the tibia, as measured by an independent observer in the immediate postoperative and 1-year follow-up X-rays, did not show any significant difference between the groups, indicating that there was no secondary loss of reduction. The mean postoperative angulation in the coronal plane (varus/valgus) was 2.7° (range 0–7°, SD = 1.98) in the IMN group and 2.1° (range 0–8°, SD = 1.77) in the PTP group; both of these tended towards a varus inclination, but there was no statistically significant difference between the groups (*p* = 0.296). In the sagittal plane, the mean extent of postoperative procurvatum/recurvatum was 2.6° (range 0–8°, SD = 1.82) in the IMN group and 2.2° (range 0–9°, SD = 1.98) in the PTP group; both of these tended towards procurvatum, but there was no statistically significant difference between the groups (*p* = 0.415). >5° of malalignment was seen in four patients (21.1 %) in the IMN group (one patient had varus and three had anterior apex deformity) and in four patients (16 %) in the PTP group (two patients had varus and two patients had procurvatum). The average range of motion was 119.7° (range 90–150°, SD = 19.18) in group A and 115.2° (range 80–150°, SD = 17.28) in group B (*p* = 0.462). There were complaints of occasional anterior knee pain and discomfort upon kneeling on the floor from six patients (31.6 %) in group A and two patients (8.0 %) in group B (*p* = 0.097).

## Discussion

Data allowing a comparison of tibial nail and minimally invasive plating for extra-articular proximal tibial fractures are scarce. The primary goal of this prospective study was to compare the results of tibial nailing and minimally invasive plating from various aspects.

In the present study, patients in the IMN group had a significantly shorter length of hospital stay compared with those in the PTP group (*p* < 0.05) because of the smaller incision made during closed nailing, meaning that IMN results in less of an economic burden and a lower cost of healthcare to society than PTP.

Although early weight-bearing is inherently associated with a load-sharing device such as an IMN, the literature does not accurately predict an accepted time at which full weight-bearing should be initiated with either procedure. Various studies have often stated that weight-bearing should be initiated when it can be tolerated by the patient [6]. In previous studies of extra-articular proximal tibial fractures treated with IMN, full weight-bearing was initiated at various times ranging from 0 to 16 weeks, depending on the fracture location, fracture pattern, and surgeon’s preference [11, 19]. Similarly, in extra-articular proximal tibial fractures treated with PLP, time to full weight-bearing has ranged from 6 to 13 weeks for the same reasons [6, 8, 18]. In our study, the time required before full weight-bearing, which was done only after complete radiological union, was significantly less in the IMN group (18.26 weeks) as compared to the PTP group (22.84 weeks). Although these times are longer than those stated in previously published reports, we started full weight-bearing only after complete clinical and radiological fracture union. That being said, we started passive and active assisted movements early—from day 2, progressing later to partial weight-bearing. Hence, we found no significant differences in range of motion of the knee between the groups.

Reported infection rates range from 0 to 8 % in nailing patients [5, 12, 17] and from 0 to 14 % in plating patients [17, 18, 20]. But, in the study by Lindvall et al. [6], the authors reported significantly higher infection rates: 28 % in the nailing group and 24 % in the plating group. The most probable reason for this is the higher proportion (42.8 %) of patients with open fractures in their study [6]. In the systemic review by Bhandari et al. [17], the infection rates were 2.5 % in the nailing group and 14 % in the plating group. The infection rates in our series were 5.3 % in the IMN group and 8 % in the PTP group (*p* = 0.738).

Malunion is a documented complication of the nailing of proximal tibia fractures and has been reported to occur in 3–100 % of cases in previous studies (Table 2) [9, 11, 17]. In our study, there was a malreduction/malunion rate of >5° in the IMN group (four patients, 21.1 %): varus malalignment in one patient and anterior apex deformity in three patients. Various techniques have been described for preventing malreduction, including the use of blocking screws [5, 6], unicortical plating [13], a universal distractor [14], nailing in the semiextended position [15], or the use of a nail with a more proximal Herzog bend [16]. In our study, we used blocking screws in three cases, reduction plating in one case, and a universal distractor in two cases. In the other cases, a reduction clamp was used to prevent proximal fragment extension while inserting the nail. A common technique employed in all of the nailing cases was to make a slightly higher entry point than that normally used for tibial nail insertion. This modification brought our insertion point more in line with the medullary canal of the tibia, hence reducing the extension of the proximal fragment. The plating group also had four cases of malunion (16 %), but the difference was not statistically significant. In a systemic review of 17 studies by Bhandari et al. [17], the authors reported a higher malunion rate in the nailing group (20 %) than in the plating group (10 %). Similarly, Lindvall et al. [6] reported a higher malunion rate in the nailing group—apex anterior malreduction occurred in 36 % of the patients in the IMN group and 15 % of those in the locking plate group—although this difference was not statistically significant.

**Table 2:** Comparison of data obtained in the present work with data presented in the literature

References	Infection		Union rate		Malunion	
	IMN group (%)	PTP group (%)	IMN group (%)	PTP group (%)	IMN group (%)	PTP group (%)
Bhandari et al. [17]	2.5	14	96.5	98	20	10
Lindvall et al. [6]	28	24	77	94	40.9	20.6
Beuhler et al. [12]	0		92.9		7.1	
Tornetta and Collons [15]	0		0		10	
Present study	5.3	8	94.7	96	21.1	16

When union rates after the initial fixation were analyzed in our study, it was found that the union rate in the IMN group was 94.7 and that in the PTP group was 96 % (*p* = 0.849). The high union rates observed in our series are consistent with those stated in various published reports, which range from 91 to 100 % [6, 8, 11, 19]. Our results were, however, higher than seen in a study performed by Lindvell et al. [6], where the authors noted union rates of 77 % in the IMN group and 94 % in the PTP group. We believe that this difference in union rates arose because open fractures were excluded from our series, not because of the type of procedure performed. The locked nail technique demonstrated advantages in terms of the operation time, hospital stay, early full weight-bearing, and time required for bony union.

We concluded from our study that intramedullary nail is superior to minimally invasive plating in terms of brevity of hospital stay and speed of union along with early full weight-bearing, but there was no clear advantage of either technique in terms of operative time, infection rate, range of motion of the knee, and rates of malunion and nonunion. Both implants yielded promising results with extra-articular proximal tibial fractures and provided rigid fixation that prevented secondary fracture collapse.

Limitations of this study include the small number of patients, the involvement of multiple surgeons, the absence of long-term follow-up to evaluate the outcome of malalignment in terms of the development of osteoarthritis of the knee, and the use of both stainless steel and titanium implants, which may affect infection rates because titanium is more biocompatible than stainless steel, meaning that using titanium reduces the soft-tissue reaction and reduces the chance of infection.
